# Home care and end-of-life hospital admissions: a retrospective interview study in English primary and secondary care

**DOI:** 10.3399/bjgp19X704561

**Published:** 2019-06-18

**Authors:** Sarah Hoare, Michael P Kelly, Stephen Barclay

**Affiliations:** Primary Care Unit, Institute of Public Health, School of Clinical Medicine, University of Cambridge, Cambridge, UK.; Primary Care Unit, Institute of Public Health, School of Clinical Medicine, University of Cambridge, Cambridge, UK.; Primary Care Unit, Institute of Public Health, School of Clinical Medicine, University of Cambridge, Cambridge, UK.

**Keywords:** end of life care, home palliative care, hospitalisation, primary care

## Abstract

**Background:**

Enabling death at home remains an important priority in end-of-life care policy. However, hospital continues to be a more prevalent place of death than home in the UK, with admissions at the end-of-life often negatively labelled. Admissions are frequently attributed to an unsuitable home environment, associated with inadequate family care provision and insufficient professional care delivery.

**Aim:**

To understand problems in professional and lay care provision that discourage death at home and lead to hospital admissions at the end of life.

**Design and setting:**

A qualitative study of admission to a large English hospital of patients close to the end of their life.

**Method:**

Retrospective in-depth semi-structured interviews with healthcare professionals (*n* = 30) and next-of-kin (*n* = 3) involved in an admission. Interviews addressed why older patients (>65 years) close to the end of life are admitted to hospital. Interviews were transcribed and analysed thematically.

**Results:**

Home-based end-of-life care appeared precarious. Hospital admission was considered by healthcare staff when there was insufficient nursing provision, or where family support, which was often extensive but under supported, was challenged. In these circumstances, home was not recognised to be a suitable place of care or death, justifying seeking care provision elsewhere.

**Conclusion:**

Challenges in home care provision led to hospital admissions. Home end-of-life care depended on substantial input from family and professional carers, both of which were under-resourced. Where either care was insufficient to meet the needs of patients, home was no longer deemed to be desirable by healthcare staff and hospital care was sought.

## INTRODUCTION

Supporting patients at the end of life to die at home and preventing their admission to hospital is a significant task for GPs and community nursing teams. Place of death is a key policy marker of end-of-life care success in both the UK^1^^,^^2^ and internationally,^3^ though recent literature challenges the priority given to location in end-of-life care.^4^ Research has demonstrated that patient preferences for place of death are complex,^5^ that notions of home are malleable and include other settings which may feel ‘home like’,^6^^–^8 and that the hospital is important in end-of-life care provision.^9^^–^11 However, death at home remains a policy concern. Reflecting this, end-of-life hospital admissions are often defined as ‘avoidable’, ‘preventable’ or ‘inappropriate’,^5^^,^^12^^,^^13^ particularly compared to community-based care.

It is not clear why patients at the end of their lives are admitted to, and die, in hospital^14^^,^^15^ though many reasons have been suggested. It seems likely that the provision of end-of-life care in the community from both professional and lay carers is an important factor, recognised in both the limited literature exploring end-of-life hospital admissions^11^^,^^14^^,^^16^^–^18 and in related research on community-based end-of-life care. For example, the availability of professional home-based end-of-life care is associated with the likelihood of patients with cancer dying at home rather than in hospital.^19^ Notably however, the relationship between professional end-of-life care in the community and hospital admissions has yet to be explored robustly.^20^ The challenge for family carers in providing care at home and the association between problems in this care and end-of-life hospital admissions is frequently identified in policy, and recognised by GPs.^11^^,^^18^^,^^21^ Problems are often attributed to family members being ‘panicked’ by changes to a patient’s symptoms in the dying phase,^22^ or because family members felt unable to cope with incessant care provision, and their own distress in watching a relative die.^23^^,^^24^ Similar to professional care however, the circumstances that lead families to seek hospital care have not been considered in detail.

Hospital continues to be a more prevalent place of death than home in England.^25^ Understanding why these admissions occur will help GPs and community nurses to support patients in their end-of-life care, whether at home or elsewhere. This article explores empirically the relationship between the care requirements of home-based end-of-life provision and hospital admissions using healthcare staff, and family carer perspectives of why patients previously cared for in the community are admitted to, and die, in the acute hospital setting.

## METHOD

### Design and participants

The data presented are from a larger study exploring end-of-life hospital admissions.^26^^,^^27^ The focus of this article is on why patients close to the end of life who are cared for at home do not die there but are instead admitted to hospital. Interviews (*n* = 33) were conducted with healthcare staff and next of kin involved in the admission to a large English hospital of a patient who subsequently died there within 3 days of admission (a ‘case-patient’).

**How this fits in**

It is known that dying at home is an end-of-life care policy priority, with reducing end-of-life hospital admissions targeted in end-of-life care improvement measures. Many people currently die in hospital. Understanding why deaths occur in hospital rather than at home is underexplored but is often assumed to be associated with problems in care provision. This study shows that home-based end-of-life care can be precarious and an absence of nursing care or family support may facilitate hospital admission; deaths in hospitals can occur where home is not considered a suitable place of care and death, and offers benefits to patients, family carers, and staff.

Interviewees included community healthcare staff (GPs, a community specialist nurse, and ambulance staff) hospital staff, and the case-patients’ next of kin. This study centres on the accounts of interviewees involved in the care of a case-patient who had been admitted to hospital from home. Additionally, the accounts of interviewees involved in the admission of case-patients from other community settings have been included as they usefully reflected on other patients admitted from home. These quotations are indicated by (a).

### Data collection

All interviews were semi-structured, in depth, and were conducted between 2012 and 2013. Healthcare staff interviews addressed the participants’ involvement in the case-patients’ admission, their views on the reasons for the admission, and end-of-life admissions generally. These interviews were conducted within a month of the interviewee’s involvement in the case-patient’s care, lasted approximately 30 minutes, and occurred at the participants’ place of work or nearby. Next-of-kin interviews considered the case-patient’s care in the last months before death, including their involvement in care provision. These interviews took place 4–7 months after the case-patient’s death, lasted approximately 1 hour, and occurred at their home.

### Analysis

Interviews with staff and next of kin provided highly detailed accounts of the circumstances that led to the hospital admission of case-patients and for staff interviewees, similar patients they had cared for previously. Interviews were audiorecorded and professionally transcribed verbatim. The transcripts were analysed thematically to understand the reasons that facilitated admission to hospital. Transcripts were coded by hand and then using the software NVivo (version 10), with sections of the text tagged using both a priori and emergent data categories. Codes were then sorted and charted to assess both the breadth and depth of data. Data were synthesised to understand both the individual case-patients’ admission, and the admissions collectively, with synthesis aided by reference to both end-of-life care and sociological literature. Extended details about the study methodology^26^^,^^27^ and related findings^10^ can be found elsewhere.

Quotations in the text are followed by the participants’ role, study number, and interview page number. Identifying details have been omitted to protect the anonymity of the interviewees and the deceased case-patients.

## RESULTS

Appendix 1 shows the characteristics of the case-patients and Table 1 provides a summary of all participant roles. Hospital admissions were instigated by patients, family carers, GPs, and ambulance staff, often working in collaboration. Dying at home was desirable for patients according to healthcare staff and next of kin. However, healthcare staff did not always feel that patients’ place of care was necessarily a suitable environment to either receive end-of-life care or die. Important factors in this evaluation were the availability of professional and family care provision, and problems in either were pertinent to the decision to facilitate patients to hospital.

**Table 1.** Study participant roles, *N* = 33

**Interviewee role and profession**	***n***	**Those involved in care of case-patients admitted from home, *n***	**Those involved in care of case-patients admitted from another setting, *n***
**Community staff**			
GP	5	4	1
Community nurse specialist	1	1	0
Ambulance staff (including paramedic, student paramedic, emergency care assistant)	6	3	3

**Hospital staff**			
A/E doctor (including consultant, registrar, foundation)	6	3	3
Ward doctor (including consultant, registrar, foundation)	11	7	4
Pastoral worker	1	1	0

**Next of kin**			
Spouse or adult child	3	2	1

A/E = accident and emergency.

### Facilitating death at home was desirable

Dying at home was advocated by healthcare staff from all occupational roles. The desirability of death at home was conceptualised using ideas of familiarity, comfort, and the presence of loved ones, as well as the fulfilment of perceived patient preferences about place of death. Even where staff articulated practical difficulties in supporting a patient’s death to occur at home, it was typical to concurrently recognise the desirability of patients dying at home:

‘Most people want to die at home right? Surrounded by, you know, their friends, family and everything else.’

(GP, 4, 5)

‘I think people should be able to die at home. Most elderly and terminal patients do not want to die outside their home but I think there is a general fear and lack of confidence by relatives etc, and I don’t think necessarily there would be resources to manage that. So I think if the resources were in place to help support the family then I do think that would be a good option.’

(GP, 10, 9)

### Importance of formal care provision

Despite the aspiration for home-based end-of-life care, the community was not always a viable or a desirable place of care for patients according to interviewees. A significant factor in this negative assessment of home was the absence of sufficient domiciliary care to address a patient’s needs. For patients deemed to be in that situation, the utility of hospital as a care provider was recognised by interviewees. Community staff described facilitating admissions to ensure patients received appropriate care:

‘I think the thing that we struggle with in the community is the resources to manage a death, sometimes it can be quite difficult for the district nurses with short notice depending on what else is on their caseload. Things like hospice, it depends on bed availability and so it can be quite frustrating, you know what you want to do or you know how you want to manage it and sometimes people do have, you know difficult symptoms that we have to refer to hospice, if they don’t have a bed then sometimes it becomes difficult because what do you do? You’re kind of caught between a rock and a hard place really.’

(GP, 15, 7)

### Availability and timeliness of nursing provision

Community nursing was well regarded by interviewees, particularly GPs. However, the limited availability of this care, particularly at short notice, was a significant problem. Staff described the challenge of maintaining patients at home where the patients’ care needs had escalated and additional care was required sooner than care could be organised or was available. This was particularly applicable when a patient’s condition had deteriorated rapidly or they were experiencing unanticipated symptoms. In these circumstances, hospital care was sought:

*‘I wondered about perhaps keeping* [the case-patient] *at home with some district nurse support* [...], *unfortunately when I rang the district nurses they were very busy with* [a] *cancer patient and there wasn’t a district nurse available to come and help, so I had a chat to the patient and* [their spouse] *and said, “what do you feel about being admitted?”, and* [the case-patient] *wasn’t that keen to go up* [to the hospital], [their spouse] *was quite keen for* [them] *to be looked after,* [the spouse] *was struggling.’*

(GP, 1, 2)

### Community care was supplemented by family care

Problems in domiciliary end-of-life care were also associated with family care provision, especially for patients resident at home. These patients often received personal care from spouses and adult children who supported their day-to-day living and healthcare needs. Where patients required more care than family members could provide, hospital admission was often sought both by community staff and family members. Staff descriptions of these admissions typically involved negative portrayals of family members as being unwilling to provide terminal care, or more charitably, as being unprepared for the patient’s death:

*‘I felt that the main problem was that the family weren’t able to manage* [the case-patient’s] *condition at home. I felt it would have been more appropriate for* [them] *to have stayed at home but to have had obviously a lot, the nursing support if it had been available at home.* [...] [but arranging additional care] *wouldn’t have necessarily changed anything because I don’t, as far as I understand I don’t think there is that service available where, you know, they would have somebody at home nursing* [them] *and I just think the family did not want to nurse* [them] *to* [their] *death.’*

(Locum GP, 10, 5)

To understand why hospital admissions may have been sought because family members did not ‘want’ to continue to provide care it is useful to explore the family caring role. In the next section, the demands on family carers’ time, physicality, and experience are examined in relation to end-of-life hospital admissions.

### Experience to provide care

Family care provision at home could be compromised by the limited experience some family carers had of death and end-of-life care. For healthcare staff, naïve family carer expectations of the dying process were thought to hinder the likelihood of death at home, as unfamiliar but typical end-of-life care symptoms were reported to lead family carers to seek reassurance and care. Consequently, hospital admissions were thought to occur, either directly, as carers sought help from ambulance or out-of-hours services, or indirectly, if they requested additional help that could realistically only be fulfilled in hospital:

*‘* [...] *So it does seem that we had talked about end-of-life care but we probably didn’t talk about preferred place of care or maybe the family weren’t prepared well enough for the last stages of* [the case-patient’s] *dying to manage with the support that we have.’*

(Ward doctor, 14, 1)

For family carers, inexperience was often expressed as concern about the quality of their care provision, particularly compared to professional carers. While this was not cited by family carers as a reason for admission — family carers who had facilitated case-patient admissions said they did so to access necessary medical support — they acknowledged the hospital to be a place for professional attention and a place where their relative would receive appropriate care:

*‘I was pleased* [they] *died in hospital, because I always used to think “If you’re ill, hospital is the best place to be, because there you can have all the correct attention and everything that you want”. At home, I mean, I don’t know all that much about the medical service really,* [...] *I couldn’t be a nurse, I would do what I could for* [my spouse] *but I don’t know, you know, how well I would be doing it compared with a qualified nurse.’*

(Next of kin, 6, 13)

### All-encompassing care

Supporting a dying relative often involved significant investment of time and resource by family carers. The consequences of this to the family carer was infrequently recognised by the family carer themselves, who typically dismissed their own care provision as just part of their family role. However, providing care could have negative effects on the family carer’s wellbeing, and healthcare staff described how a patient’s care need could exceed the family carer’s capacity to provide care. In these instances, additional care was often recognised to be necessary, whether sought from hospital providers or elsewhere:

*‘I don’t think* [the family] *understood how much work it would be* [caring for the case-patient]. [...] *They just looked drained. I think, maybe, I don’t know why they never had any care.* [...] *They were doing* [the care] *all themselves. The daughter was living there and she was doing, getting up in the night and* [...] *I think* [the case-patient’s] *partner* [too]. [...] [A]*nd the daughter just looked so, so tired.* [...] *And I, I mean, maybe with a bit more ... they was saying to me about* [the case-patient] *going into, maybe a home. They understood that* [the case-patient] *had probably got to that point because* [the case-patient] *was getting very difficult for them to manage.* [...] *Because* [the case-patient] *didn’t sleep much at night, the family was all up all night and then they were finding it difficult during the day.’*

(Specialist nurse, 11, 3–4).

### Physical care

The family caring role was recognised by professional staff to be physically demanding. This could make family care provision difficult, particularly if a carer was a frail older individual and the patients’ health had deteriorated such that they required substantial hands-on support. In these instances, both community and hospital interviewees recognised that the continued maintenance of patients at home was impractical without additional support. Where this was not thought possible, staff recognised the helpfulness of care elsewhere, including hospital:

*‘And there is, you know, this practical side of nursing people, you know, mobilising, even moving them safely when they can no longer move themselves.* [...] *I went to visit an elderly couple and found both of them on the floor where they had been, God help them, all that night, because she had been trying to get him out of bed to the loo and he had fallen on top of her, and there they had lain all night long.’*

(aGP, 28, 10)

‘[...] *the factors that contributed* [to the case-patient’s admission] *were an inability to at that time ensure* [they were] *comfortable at home, uncertainty about how best to meet* [their] *needs, lack of nursing support at home because* [they] *were there with* [only their partner]. *It’s a little bit difficult to see how anything else could have happened unless a nursing team could have been on site or a doctor and then perhaps with a nurse could have been on site to support* [their] *final hours. Part of that is my ignorance, I don’t think I know enough about how you support a death to happen while at home when you just have, say, an elderly spouse there.*’

(Ward doctor 5, 3–4)

### Caring for patients at home

Ensuring a patient could die at home in a manner that met staff expectations of an appropriate death was not easy. The factors identified above were often considered to be interdependent issues, which could be challenging for GPs to meet and therefore could precipitate hospital admissions:

*‘And nursing someone who is ... is ... you know, completely physically dependent, it’s very strenuous, and you need a lot of kit. And* [...] *even the changes that you have to ... you know, it’s ...* [...] *We* [GPs] *like people to die at home, and we do try very hard to organise it as much as we can. But it’s ... it’s difficult. I mean, the* [community care team] *are amazing. I wish there was about eight times more of them.’*

(aGP, 28, 10)

## DISCUSSION

### Summary

The present research identifies that hospital admissions at the end of life can be a consequence of the challenges in delivering end-of-life care at home. Insufficient available nursing provision and family carers who had exceeded their capacity to care countered the desirability of home as a place to deliver end-of-life care. These circumstances often caused GPs, together with family carers and other healthcare staff to seek care for patients through hospital admissions.

### Strengths and limitations

This article has explored the consequences of insufficient community-based care as a precursor to end-of-life hospital admissions. This research usefully contributes to the limited evidence base on end-of-life hospital admissions. In a policy and clinical context where the desirability of dying at home is presumed, the present findings also add insight to the practicalities of home-based end-of-life care.

The findings of this study are from an in-depth qualitative study of 33 healthcare professionals and next of kin involved in the admission to one English hospital of nine patients close to the end of life. The extent to which interviewee accounts of the case-patients’, and similar patients’, community-based care, and the reasons for their hospital admission, can be extrapolated to end-of-life care in general is therefore limited. For instance, it is likely that some other patients with problems in the provision of professional or family care would continue to remain at home and not seek hospital care. However, the challenges faced by healthcare staff and family carers in providing end-of-life care accords with existing research, as outlined later on, and contributes to an understanding of why admissions occur.

Formal care provision that occurs at the interface of health and social care, such as supporting personal hygiene, or eating and drinking, has not been addressed in this article. This care is pertinent to understanding how end-of-life care can be facilitated at home,^28^ and paid carers were known to have supported some of the case-patients. Interviewing these carers was outside the study scope, and their role was not sufficiently elaborated by other interviewees to be considered.

### Comparison with existing literature

The limited availability and capacity of domiciliary nursing care was cited as a reason why interviewees instigated hospital admissions for patients close to the end of life. The challenge for GPs in arranging community nursing end-of-life care is also reported in a UK-wide survey,^29^ and seems likely to be due to the restricted capacity of community nursing, where rising demand for services coincides with staff shortages.^30^ Internationally, the importance of community-based end-of-life care provision for facilitating death at home accords with review evidence, which identifies receipt of community-based nursing, home palliative care, and GP home visits as key factors.^31^^,^^32^

The importance of family care provision in facilitating death at home is well-established.^31^^,^^33^ The present research shows that where lay care is recognised to be no longer tenable, healthcare providers may seek hospital care for patients, supporting GP accounts about the reasons for end-of-life admissions^18^ and pejorative rhetoric about family carers.^6^^,^^18^ However, it would be inaccurate to assert on the basis of this that problems in lay care provision instigate these admissions,^23^^,^^34^ without acknowledging simultaneously the contribution of family care provision in maintaining patients at home. Interviewees demonstrated that family members delivering hands-on care provided vital support, supplementing professional care provision.^35^^,^^36^ Therefore, it is plausible that hands-on care from family members can defer admission to hospital for some patients while resident at home. This is pertinent given the context of lay care provision: family carers were understood to be often ill-prepared for their role^1^^,^^18^^,^^34^^,^^37^ with often substantial, if typical, psychological^23^^,^^34^ and physical^6^^,^^36^^,^^38^ demands made of them, particularly for those with limited mobility such as frail older people. It seems likely therefore that family carers’ ability to sustain their care provision, and prevent hospital admissions, is compromised without supplementary support.^39^

Facilitating end-of-life care at home was important for professional carers, who echoed prevalent notions about the desirability of death at home.^8^^,^^37^ However, interviewees also recognised that the safety of end-of-life care at home could be compromised.^8^^,^^10^^,^^40^ Where the cause of this could not be addressed adequately, healthcare staff and family carers sought care for the patients elsewhere. While hospice and residential nursing homes were considered, challenges in accessing these institutions at short notice meant hospital care was prioritised^41^ highlighting the importance of hospital as a provider for end-of-life care.^10^

### Implications for research and practice

The present analysis was informed by a sociological interest in dying at home, focusing on the physical and technical infrastructure of home and hospital, the skills of lay carers and professionals, and the way dying is understood by those involved.^42^ This perspective exposed the vulnerability of home care for some patients, which can be dependent on stretched professional and lay provision, and builds on over a decade of sociological scholarship on the role of home as a place of care.^6^

The case-patients’ hospital admissions demonstrate the difficulty of providing and maintaining care at home. The authors’ findings challenge the prevalent discourse that home is an inherently better place to receive end-of-life care than hospital and highlight the dissonance between policy rhetoric and the everyday reality of caring for patients close to the end of life. The present research suggests that if policy and practice maintain an emphasis on facilitating deaths at home, there must also be a concurrent focus on ensuring that patients can die there safely. Identifying how best to achieve this will require further research, and is likely to require investment to ensure that community nursing provision is adequately staffed, responsive, and available throughout the day and night.^43^ Supplementing this essential provision with rapid and reliable specialist clinical services that offer support for patients at home for extended periods of time, such as hospice at home and Marie Curie nursing services, would also be pertinent.^44^^–^46 To facilitate care delivery, primary care clinicians must be supported by adequate information-sharing practices,^47^ and be confident in delivering palliative and end-of-life care.^48^ Family carers must also be supported, including greater recognition of their needs.^49^ Pejorative assessments of the association between family care and end-of-life hospital admissions obscures the significant undertaking of lay carers to support patients at home. Future research could productively address how families persist in providing care, and how GPs and others can best support them.

## Appendix 1. Case-patient characteristics, *N* = 9

**Table:**

**Characteristics**	***n***
**Sex**	
Female	7
Male	2
**Age, years**	
65–69	1
70–79	2
80–89	5
>89	1
**Condition**	
Cancer	2
COPD	2
Dementia	5
**Previous place of care**	
Home	6
Residential home^a^	3
**Time of admission**	
Weekday (9.00 am to 5.00 pm)	3
Weekday (5.00 pm to 9.00 am)	2
Weekend	4

a Residential home refers to both nursing and care homes. COPD = chronic obstructive pulmonary disease.
